# Depression in newly diagnosed type 2 diabetes

**DOI:** 10.4103/0973-3930.62601

**Published:** 2010

**Authors:** Ruan Yu, Li Y-Hua, Li Hong

**Affiliations:** 1 Department of Endocrinology of SIR RUN RUN SHAW Hospital, School of Medicine, Zhejiang University, SIR RUN RUN SHAW institute of Clinical Medicine of Zhejiang University, Hangzhou, Zhejiang - 310 016, China; 2 MISHIXIANG Community Health Service Center, Hangzhou, Zhejiang - 310 011, China; 3 Department of Endocrinology of SIR RUN RUN SHAW Hospital, School of Medicine, Zhejiang University, SIR RUN RUN SHAW institute of Clinical Medicine of Zhejiang University, Hangzhou, Zhejiang - 310 016, China

**Keywords:** Diabetes, depression, erectile dysfunction

## Abstract

**Objective:**

To investigate the incidence of depression in newly diagnosed type 2 diabetes. Materials and

**Methods:**

One hundred newly diagnosed (4 – 12 weeks) T2DM participants were evaluated for depressive symptoms by using the Self-Rating Depression Scale (SDS). Blood glucose (HBA1C), urinary albumin, BMI, and blood pressure were measured. Sexual function was evaluated by a self-score on IIEF-5 Questionnaires in male participants below 60 years of age.

**Results:**

Twenty-eight (28%) of these had depressive scores, 18 (18%) had mild scores, six (6%) had moderate scores, and four had (4%) severe ones. In those who took oral medication, the percentage of depression was 18.5% (10/54) and in those who were treated by insulin the percentage was 39.1% (18/46). The levels of fasting blood glucose (FBG), HBA1c, and urinary albumin were higher in those with depression. The SDS score was negatively correlated with age and annual household income (r = 0.151,0.139, *P* < 0.05); 17% of the (8/48) males below 60 years of age was diagnosed with erectile dysfunction (ED) by II EF-5score < 20 and the severity of depressive symptoms was negatively correlated with II EF-5score (*r* = 0.131, *P* < 0.05).

**Conclusion:**

These findings indicated that depressive symptoms are common in newly diagnosed type 2 diabetics. A variety of factors could be influencing the severity of depressive symptoms.

## Introduction

Type 2 diabetes is a chronic disease; individuals with diabetes are at risk of complications, such as, nephropathy, retinopathy, and cardiovascular complications. Additionally, a long duration of strict diet limit, glucose monitoring,[1] and medication can cause psychological maladjustment, studies have shown depressive symptoms to be higher in individuals with diabetes[2,3] and the prevalence goes up with disease duration and complications.[4] Studies have shown the percentage of depressive symptoms in newly diagnosed diabetes.

We have investigated 100 newly diagnosed T2DM from January to July of 2008, for the incidence of depression.

## Materials and Methods

### Subjects

One hundred participants were recruited from the Outpatient Clinic. Informed consent was acquired from them. They were diagnosed with T2DM with the help of the 1999 WHO criteria. The time of diagnosis was between 4 to 12 weeks. The subject population comprised of 60 males and 40 females, the average age was between 49 ± 11 years, and all were married. Fifty-four participants were treated with oral anti-diabetic drugs and 46 participants were treated with insulin. All the participants had no past psychotic disorders or family history, no other severe chronic disease or acute stress such as infection.

### Methods

Body height, weight, and blood pressure of every participant were measured and BMI was calculated. Blood glucose, glycosylated hemoglobin A_1c_ (HbA_1c_), and urinary albumin test were performed. Zungs' Self-Rating Depression Scale (SDS) was used to screen the participants for depressive symptoms. The SDS is a self-rating scale containing 20 questions about depressive symptoms. We used the Chinese version of the SDS, which was translated previously. Each item was anchored on a four-point scale (1 = no to 4 = always). In SDS, an adjusted score of less than 49 points was regarded as indicating a normal state, and 50 to 59 points, 60 to 69 points, and over 70 points indicated mild, moderate, and severe depressive symptoms, respectively. Related information, such as, annual household income, insulin dosage, oral medication varieties, and educational background was recorded. Sexual functions of 48 males below 60 years age were evaluated by the five-item version of the International Index of Erectile Function scale (II EF-5). Erectile dysfunction could be diagnosed when the score of II EF-5 was less than 20.

### Statistics Analysis

Data were analyzed with *SPSS* 11.5. *T-*test was performed for comparison between groups. Spearman's Correlation analysis was used to assess the relationship between SDS score and each possible correlation factor.

## Results

There were 28 (28%) participants who had depressive scores, 18(18%) were mild, six (6%) were moderate, and four (4%) were severe. Males 26% (16/60) and females 30% (12/40) could be diagnosed with depression. In those who took oral medications, the percentage of depression was 18.5% (10/54) and in those who were treated with insulin the percentage was 39.1% (18/46).

Table 1 shows the comparison between those participants with and without depression and the levels of fasting blood glucose (FBG); HBA_1C_ and urinary albumin were higher in those with depression (*P*<0.05). The difference of BMI and systolic blood pressure (SBP) between the two groups was not statistically significant.

**Table 1:** Comparison of data between patients with and without depression (X ± s)

Group	N	BMI Kg/m^2^	FBS mmol/1	HBA1C %	Urinary albumin mg/24h	SBP mmHg
Without depression	72	23.9 ± 1.6	7.9 ± 1.3	7.5 ± 0.2	20 ± 12	125 ± 11
With depression	28	23.7 ± 1.8	9.1 ± 1.6	8.5 ± 0.3	48 ± 22	127 ± 9

Group with depression versus group without depression *P < 0.05, **P < 0.01

The educational background, insulin dosage, and oral medication varieties against SDS scores using the Spearman's correlation coefficient showed no statistical significance. The SDS score was negatively correlated with age and annual household income, respectively, r = 0.151, 0.139, *P* < 0.05). Seventeen percent (8/48) of the males below 60 years age were diagnosed with ED by II EF-5 score <20 and the severity of depressive symptoms was negatively correlated with an II EF-5 score of (r = 0.131, *P* < 0.05).

## Discussion

Numerous studies have addressed the risk of depression developing in patients with medical illnesses. According to the World Health Organization (WHO) Collaborative Study, patients with diabetes have been shown to have significantly more depressive symptoms. Newly diagnosed T2DM are the specific population who suffer from not only somatic symptoms of the disease, but indisposition due to lifestyle alteration, which can possibly cause depressive emotions on the initial stage of diagnosis. Negative emotions can cause stress in diabetes patients, while a series of adverse hormones increase, they reduce the sensitivity of insulin and induce resistance to insulin.[5] Negative emotions can cause harm to the glucose control or quality of life and should be detected early to be intervened.[6]

The Self-Rating Depression Scale (SDS) was made up of self-questionnaires, which could efficiently evaluate the severity and changes of depressive symptoms; the time span for evaluation was the recent one week. Our study showed that in 100 newly diagnosed T2DM, 28% participants could be diagnosed with depression in different degrees, higher than the prevalence in the common population.[7] The results suggested that the depressive tendency was obvious and should be recognized by healthcare and family. Women had more depressive symptoms than men in some studies,[8] while our results showed 30% women and a little more than 26% men showed depressive symptoms. Our findings agreed with the previous studies.[9,10] The severity of chronic disease had an effect on the onset of depression. We also found the incidence of depression greatly increased in those treated with insulin, however, the severity of depressive symptoms was not relevant to the dosage of insulin, which indicated that the anxiety of an injection of insulin could be a major factor that caused depression; therefore, in some newly diagnosed patients, the essentiality of aggressive and intensive treatment should be questioned.[11]

In addition, in developing countries, the SDS score was negatively correlated with age and annual household income,[12] which indicated some social factors, such as, incomplete medical insurance or insufficient social welfare, which could influence the incidence of depression. Incidence of ED is 35%-75%, in diabetes, was relevant to microvascular complication and impairment of autonomic nerve,[13] whereas, some functional ED was relevant to depression or anxiety and could be rectified by remission of the negative emotion. ED could be detected clinically using the International Index of Erectile Function (IIEF) questionnaire, which had been extensively used; the sensitivity and specificity were high. In our investigation, there were 17% (8/48) men who could be regarded as ED and the severity of depressive symptoms was relevant to II EF-5score. ED and depression perhaps make up a vicious cycle and damage the quality of life.[14]

The SDS was a short-range, self-questionnaire and could be re-evaluated for detecting change of depression for a second time after comprehensive treatment and psycho-intervention.

A complete realization of emotional disorder is important for better recovery of physical and mental health in newly diagnosed diabetes.[15]
